# SOX-2 and EZH-2 Expression in Primary Epithelial Malignant Salivary Gland Tumors

**DOI:** 10.3390/medsci14020188

**Published:** 2026-04-09

**Authors:** Constantin Aleodor Costin, Adriana Grigoraș, Cornelia Amalinei

**Affiliations:** 1 Department of Morphofunctional Sciences I, Grigore T. Popa University of Medicine and Pharmacy Iasi, 700115 Iași, Romania; aleodor.costin@umfiasi.ro (C.A.C.); cornelia.amalinei@umfiasi.ro (C.A.); 2 Institute of Legal Medicine Iasi, 700455 Iași, Romania

**Keywords:** SOX-2, EZH-2, primary epithelial malignant salivary glands tumors, therapy, prognosis, survival

## Abstract

Background: Malignant salivary gland tumors represent a highly diverse group of neoplasms, their heterogeneity likely arising due to variable origin in different tissue components. Emerging evidence suggests that SOX-2 and EZH-2 play critical roles in salivary gland carcinogenesis, being related to tumor cell stemness potential, along with accelerated tumor progression and unfavorable clinical outcomes. The aim of this study was to assess the association between SOX-2 and EZH-2 expression, survival parameters, and tumors’ pathological characteristics in a group of patients with primary epithelial malignant salivary gland tumors (MSGTs) and to evaluate their value as diagnostic and prognostic markers. Methods: Our study group comprised 104 patients with primary epithelial MSGTs diagnosed in “Sf. Spiridon” County Hospital, Iasi, over a period of fifteen years. Pathological parameters and survival evaluation, along with SOX-2 and EZH-2 immunohistochemistry assessment and scoring, were conducted, and the associations between different parameters were analyzed. Results: High SOX-2 immunoexpression was significantly associated with lymphatic invasion (LY) (*p* = 0.003), pT stage (*p* = 0.010), histological tumor type (*p* = 0.003), and tumor grading (*p* = 0.037), while high EZH-2 immunoexpression was significantly associated with perineural invasion (PnI) (*p* < 0.001), vascular invasion (*p* = 0.038), LY (*p* = 0.001), tumor grading (*p* = 0.002), and pathological extranodal extension (pENE) (*p* = 0.018). The tumors with high SOX-2 and EZH-2 expressions were associated with a reduced overall survival (OS) (*p* = 0.013 and *p* = 0.011). Cox regression analysis revealed that pT (HR = 1.826, *p* = 0.019), LY (HR = 0.318, *p* = 0.007), and tumor grade (HR = 0.505, *p* = 0.021) added to high SOX-2 and EZH-2 immunoexpression independently predicted a poor survival outcome (HR = 2.373, *p* = 0.016 and HR = 2.746, *p* = 0.015). Conclusions: Our findings suggest that SOX-2 and EZH-2 may serve as biomarkers of aggressive behavior and a poor prognosis in primary epithelial MSGTs, providing potential opportunities for precision-targeted therapies.

## 1. Introduction

Malignant salivary gland tumors (MSGTs) account for only 5–8.5% of all head and neck tumors, being considered rare neoplasms in the general population [1]. Their global incidence ranges between 0.57 and 0.69 cases per 100,000 people per year, with an estimated 53,583 new cases registered in 2020 [2,3]. The reported incidence of MSGTs is around 1.1 cases per 100,000 individuals in the United States [4]. Similarly, the crude incidence rate of these tumors is 1.3 per 100,000 people per year, and the age-adjusted rate is 0.67 per 100,000 people per year, with 9917 new registered cases in Europe in 2020 [2]. MSGTs exhibit a male predominance and primarily involve the major salivary glands, mainly the parotid gland [3]. In support of this consideration, 32.6% of malignant lesions have been located in the parotid glands, followed by the submandibular glands (10.0%) and the buccal glands (9.2%), according to a recent study performed on a large cohort of patients of a southern Poland medical center [5].

Only 23% of MSGTs occurred in the minor salivary glands, registering 0.4 cases per 100,000 people between 2000 and 2007, according to the RARECARENet project data [2,3]. Additionally, a report covering a 26-year period of time showed that 61.7% of tumors located in the minor salivary glands were malignant [5].

Mucoepidermoid carcinoma is the most common histological type, followed by adenoid cystic carcinoma and acinic cell carcinoma [4]. However, their incidence may be variable across different geographical areas, considering that adenoid cystic carcinoma is most frequently diagnosed in Brazil, Turkey, Poland, and Croatia [3,5].

Despite their rarity, MSGTs are associated with a high mortality rate of approximately 40% [1]. As a consequence, intensive research has been conducted over the past decades to elucidate the molecular mechanisms involved in salivary gland carcinogenesis and to identify salivary gland malignancies’ novel diagnostic and prognostic biomarkers.

Among the emerging biomarker candidates, SOX-2 has been identified as a new potential diagnostic and prognostic marker in different cancers, including in MSGTs [6,7,8]. Regarding its characteristics, SOX-2 belongs to the sex-determining region Y-related high-mobility group (HMG)-box (SOX) family, and it is encoded by a gene located on chromosome 3q26.3–q27 [8]. SOX-2 is a key pluripotency transcription factor that plays a critical role in the salivary gland’s development, particularly in the acinar cell lineage, but it also contributes to tumor development by promoting cancer cell survival, stemness, metastasis, and drug resistance [8,9,10]. Additionally, SOX-2 may modulate cancer cell apoptosis as well as their invasive and migratory abilities by PD-L1, Wnt/β-catenin, and MAPK/c-Jun N-terminal kinase (JNK) pathway activation [7,8,11,12]. Information regarding SOX-2 expression in MSGTs is limited and sometimes controversial; however, its overexpression has been associated with tumor growth, metastasis, and poor clinical outcome [6,13].

Another promising candidate marker of MSGTs diagnosis and prognosis is the enhancer of zeste homolog 2 (EZH-2) encoded by a gene located on chromosome 7q35 [14,15,16,17]. EZH-2 acts as the catalytic subunit of Polycomb repressive complex 2 (PRC2), regulating Hox genes and initiating X chromosome inactivation by mediation of H3K27 trimethylation (H3K27me3), leading to chromatin inactivation and gene silencing [15,16,17]. EZH-2 overexpression has been consistently associated with an aberrant tumor cell proliferation and increased migratory and invasive abilities in different types of cancers, such as melanoma, along with endometrial, colon, and lung cancer [15,16]. EZH-2 is also considered a potential factor that stimulates epidermal cancer stem cells and tumor development by supporting the expression of the stemness-associated marker SOX-2 [18]. Additionally, its enhanced expression has been correlated to a poor prognosis in the squamous cell carcinoma of the head and neck [19]. Although the available data regarding EZH-2’s role in salivary gland development and tumor progression are scarce, EZH-2 is mainly correlated with an aggressive tumor profile [14,20] and with resistance to androgen receptor-targeted therapy in patients diagnosed with salivary duct carcinoma [21]. EZH-2 inhibitors have been successfully tested in oral squamous cell carcinoma [22]. Moreover, GSK343, an EZH-2 inhibitor, may inhibit SOX-2-mediated transcriptional programs that drive lung cancer cell stemness and growth [17]. Therefore, the associated tumor cell expression of SOX-2 and EZH-2 may contribute to the acquisition of an aggressive phenotype during cancer progression, both molecules being potential novel biomarkers of prognosis and therapeutic targets in different types of cancers, including MSGTs.

Considering all this accumulated data, this exploratory study aimed to assess the SOX-2 and EZH-2 expression in association with the pathological features and the survival parameters, in a group of patients with primary epithelial MSGTs, along with the evaluation of their potential role as diagnostic and prognostic markers.

## 2. Materials and Methods

### 2.1. Patients and Tissue Samples

The study group comprised 104 patients with primary epithelial MSGTs diagnosed following surgical resection, in the Pathology Department of “Sf. Spiridon” County Hospital, Iasi, between 2010 and 2024. All the participants had signed a written informed consent required for enrolment in this study. The study had received the approval of the institutional review board of the hospital (approval no. 99/8 November 2024) and of the Ethics Committee of Grigore T. Popa University of Medicine and Pharmacy Iași (approval no. 500/30 November 2024). The research adhered to the principles outlined in the Declaration of Helsinki for research involving human subjects.

**Figure 1:**
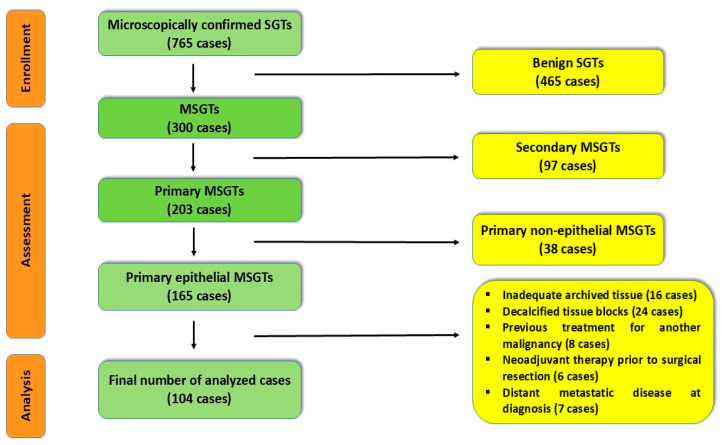
STROBE flow diagram for the confirmed cases of primary epithelial malignant salivary gland tumors. MSGTs—malignant salivary gland tumors; SGTs—salivary gland tumors.

Inclusion criteria comprised histopathologically confirmed diagnosis of primary epithelial MSGTs, along with well-preserved formalin-fixed paraffin-embedded (FFPE) tissue blocks and complete pathological and demographic information. The applied exclusion criteria were the following: inadequate archived tissue, decalcified tissue blocks, benign or non-epithelial benign salivary gland tumors, secondaries, neoadjuvant therapy prior to surgical resection, previous treatment for another malignancy, and distant metastatic disease at diagnosis (Figure 1).

The anthropometric data were collected at the time of the patient’s hospital admission. According to the World Health Organization (WHO) classification, body mass index (BMI), calculated as kg/m^2^, was used to classify the patient study group into the following categories: normal weight (BMI < 25 kg/m^2^), overweight (BMI = 25–30 kg/m^2^), and obesity (BMI > 30 kg/m^2^) [23]. Regarding the smoking status, the patients were classified as non-smokers (patients who never smoked or smoked fewer than 100 cigarettes in their lifetime), former smokers (patients who previously smoked but were not currently smoking at the time of admission), and current smokers (patients smoking at the time of assessment) [23]. Regular alcohol consumption was defined as drinking alcohol more than four days per week [23].

Surgical management was individualized according to the anatomical site and tumor extension and included the surgical approach, such as partial or total parotidectomy, wide local excision, palatectomy or infrastructure maxillectomy, hemiglossectomy, and submandibular or sublingual gland resection. More radical procedures, including facial nerve sacrifice, were performed in selected locally advanced parotid tumors.

Tissue samples collected from all patients following surgical resection were processed according to the standard histopathological examination protocols. Considering the relatively long period of time of tissue collection, histopathological re-evaluation was performed according to the latest WHO Classification and American Joint Committee on Cancer (AJCC), version 9 [24,25]. The regional lymph node metastasis (LMN), the pathological extranodal extension (pENE), and the extraparenchymal extension (EPE) were also registered [25]. According to AJCC recommendations, LNM was defined as follows: pNx (regional LNM cannot be assessed), pN0 (no regional LMN), pN1 (1–3 regional LNMs without definitive pathological extranodal extension—pENE), and pN2 (>3 regional LMNs or metastasis in any regional LMN with pENE) [25]. pENE, considered as the tumor extension through the capsules of the lymph nodes, penetrating into the surrounding connective tissue, was considered as positive or negative. Positive pENE was divided into pENE_mi_ (minor ENE ≤ 2 mm, only microscopically detectable) and pENE_ma_ (major ENE > 2 mm or macroscopic extranodal extension). The residual tumor after the surgical resection was defined as: R0 (complete resection or no residual tumor or negative microscopic margins), R1 (microscopically positive margin), and R2 (grossly positive margin or macroscopic residual tumor) [25].

Overall survival (OS) was considered to be the interval of time between the date of diagnosis and the death date or the last follow-up, whichever of these events had first occurred [26]. The last patent follow-up was on 31 December 2024.

### 2.2. Immunohistochemical Method

Representative paraffin-embedded blocks were selected in each case for immunohistochemical examination, based on the morphological features identified during the microscopic evaluation. The sections were deparaffinized in xylene and subsequently rehydrated through successive baths of decreasing alcohol concentrations. Antigenic epitope unmasking was performed using the heat-induced epitope retrieval (HIER) method, using a pH 9 retrieval solution at 95–98 °C, for 20 min. The endogenous peroxidase blocking was achieved by incubating tissue sections with 200 μL 3% hydrogen peroxide for 10 min. Incubation with anti-SOX-2 (rabbit polyclonal antibody, ab97759, dilution 1:600, Abcam, Cambridge, MA, USA) and anti-EZH-2 (mouse monoclonal antibody, ab283270, dilution 1:200, Abcam, Cambridge, MA, USA) primary antibodies was performed overnight, at 4 °C. The reaction was developed using a compatible detection system (Abcam, Cambridge, MA, USA, code ab64264) and 3,3′-diaminobenzidine tetrahydrochloride (DAB) solution for 5–10 min at room temperature. Mayer’s hematoxylin staining was used as a counterstain, followed by dehydration and mounting. The tonsillar stratified epithelial tissue was used as the external positive control, while the omission of the primary antibody during the staining procedure was used as the negative control.

### 2.3. Evaluation of SOX-2 and EZH-2 Immunoexpression

Assessment of the immunostaining pattern and extent of SOX-2 and EZH-2 nuclear immunoexpression was independently performed by two pathologists with extensive professional experience (C.A. and G.A), based on the percentage of positive cells and the staining intensity, according to the previously published SOX-2 scoring systems [6] and EZH-2 evaluation method [27,28] (Table 1 and Table 2). The differences in the assessment results were resolved by consensus.

SOX-2 and EZH-2 immunoexpressions were assessed using the multiplication of intensity and percentage of stained salivary gland tumor cells score. Using the staining pattern and its extent, SOX-2 and EZH-2 immunoreactions were scored from 0 to 9, leading to the following scoring categories: low SOX-2 or EZH-2 expressions, including scores of 0–4, and high SOX-2 or EZH-2 expressions, including scores of ≥6 (Table 1 and Table 2).

### 2.4. Statistical Analysis

Data were statistically analyzed using SPSS version 25 (IBM, Armonk, NY, USA) and Microsoft Excel 2016 (Microsoft, Redmond, WA, USA) programs. Continuous variable types were reported as mean ± standard deviation (SD). The distribution of continuous variables was analyzed using the Kolmogorov–Smirnov test. The Pearson chi-square test was used to assess the association between histopathological parameters and SOX-2 and EZH-2 expressions. The strength of the statistically significant associations was evaluated by Cramer’s V test, interpreted according to the conventional criteria (weak = 0.10, moderate = 0.30, and strong > 0.50). Survival analysis was performed using the Kaplan–Meier method, and the log-rank (Mantel–Cox) test was used to assess the differences. The immunohistochemical parameters’ prognostic significance was determined by Cox proportional hazard models, while a *p*-value < 0.05 was set as statistically significant.

## 3. Results

### 3.1. Baseline Data

A slight predominance of men compared to women (51.93% vs. 48.07%, respectively) was registered in our study group, with a men-to-women ratio of 1.08:1. The mean patients’ age group was 59.86 ± 14.91 years, with 58.20 ± 15.59 years in men and 61.64 ± 14.06 years in women. The median age of patients was 63 ± 50 years (range: 17–87). The most frequent diagnoses were registered in the 7th decade, with 30.77% of cases, followed by the 8th decade of life, with 25.97% of cases, and the 6th decade of life, with 13.47% of cases.

According to their anatomical distribution, primary epithelial MSGTs were predominantly located in the parotid glands (*n* = 49; 47.11%), followed by palatine glands (*n* = 22; 21.15%). Minor salivary gland involvement by tumors was recorded in 35.5% of cases. The tumor size ranged between 7 and 85 mm, with a mean of 36.53 ± 17.41 mm. All MSGTs were unilateral and exhibited a multifocal feature in only two cases (1.92%), both located in the parotid glands.

**Figure 2:**
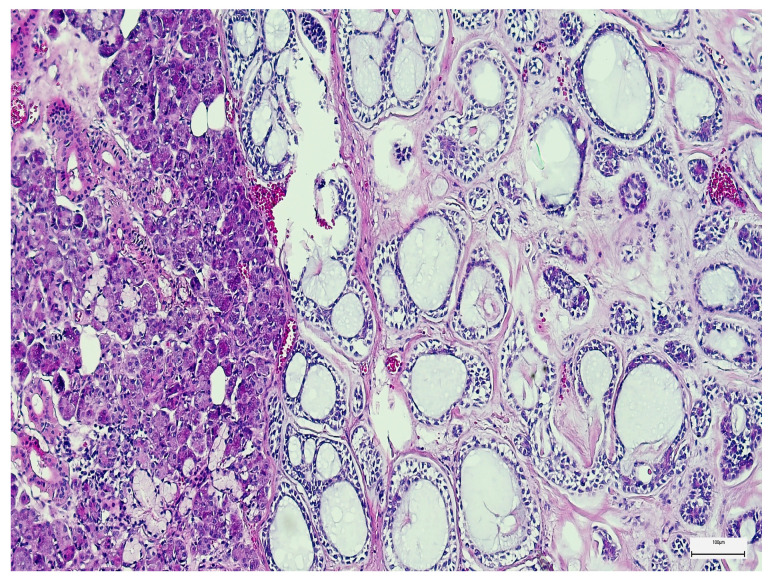
General overview of a salivary gland adenoid cystic carcinoma (H&E, 40×).

**Figure 3:**
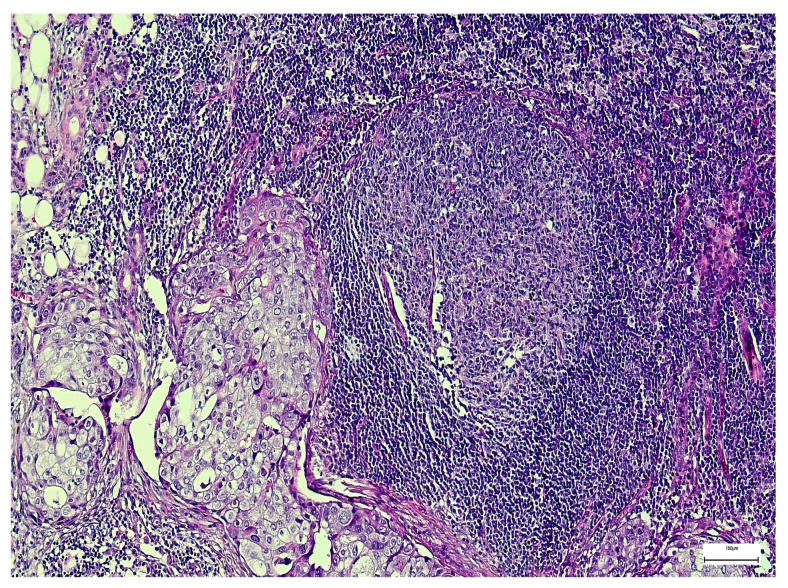
Lymph node metastasis in a salivary gland mucoepidermoid carcinoma (H&E, 100×).

The most common primary epithelial MSGT histological types were mucoepidermoid carcinoma and adenoid cystic carcinoma (*n* = 30; 28.84% of each type), while adenocarcinoma not otherwise specified (NOS) was less frequently diagnosed (*n* = 11; 10.60%) (Figure 2). Tumor grading was low in 45 cases (43.27%) and high in 34 cases (32.70%). The majority of patients presented with T2 (*n* = 34; 32.70%) or T3 disease (*n* = 39; 37.50%), and regional lymph node metastases were identified in 54 patients (51.92%) (Figure 3) by counting 189 lymph nodes involved out of 535 microscopically examined lymph nodes.

**Figure 4:**
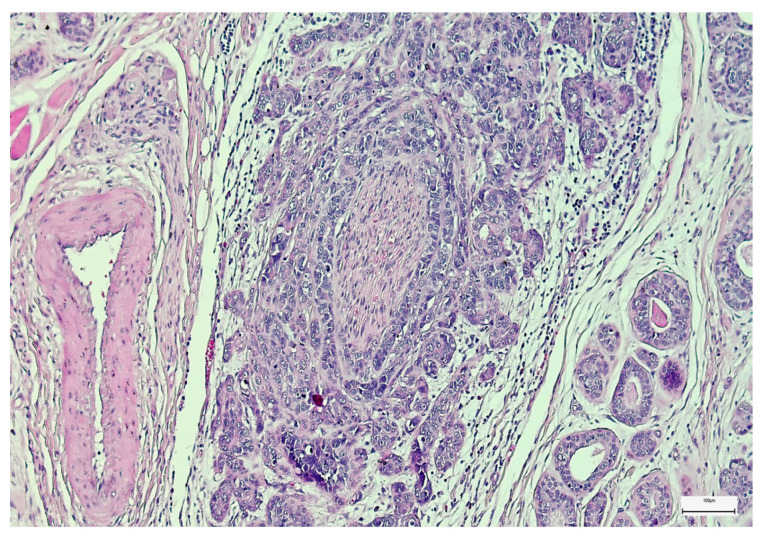
Perineural invasion in a salivary gland adenoid cystic carcinoma (H&E, 100×).

**Figure 5:**
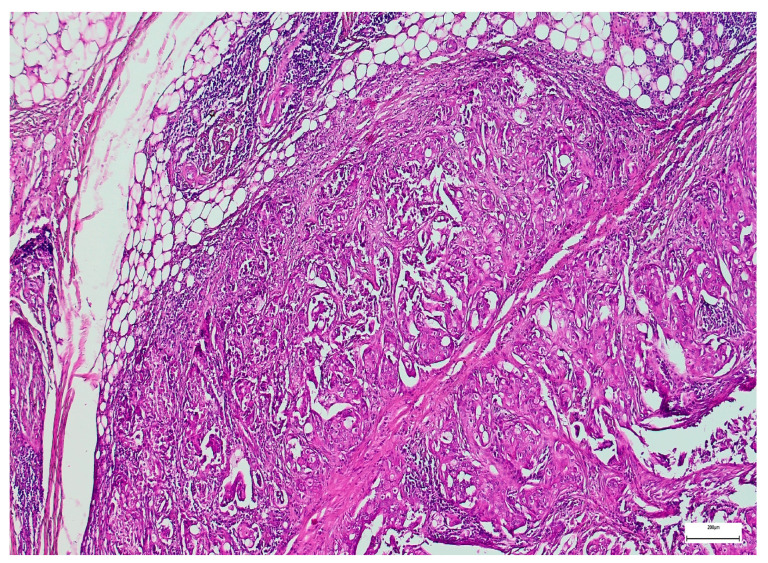
Extraparenchymal extension in a salivary duct carcinoma (H&E, 40×).

Vascular invasion (VI) was detected in 37 cases (35.58%), while lymphatic and perineural invasions were registered in 43 (41.35%) and 61 cases (58.56%), respectively (Figure 4). Microscopically positive margins after surgical resection were identified in 43 cases (41.35%), and grossly positive margins were detected in 14 cases (13.47%). Regarding EPE and pENE, a significant proportion of lesions were registered as EPE negative, while positive EPE was less common (*n* = 64; 61.53% vs. *n* = 40; 38.47%) (Figure 5). Moreover, positive pENE was detected in 23 cases (22.11%), 16 cases of them (15.38%) being diagnosed as pENE_mi_ and eight cases of them (7.69%) being diagnosed as pENE_ma_.

Regarding the therapy regimen, 57 patients (54.80%) received postoperative radiotherapy. At the time of the patients’ enrolment, most of the patients were survivors (70.20%), while 29.80% were non-survivors (Table 3).

### 3.2. Qualitative and Semi-Quantitative Assessment of SOX-2 and EZH-2 Immunoexpression

**Figure 6:**
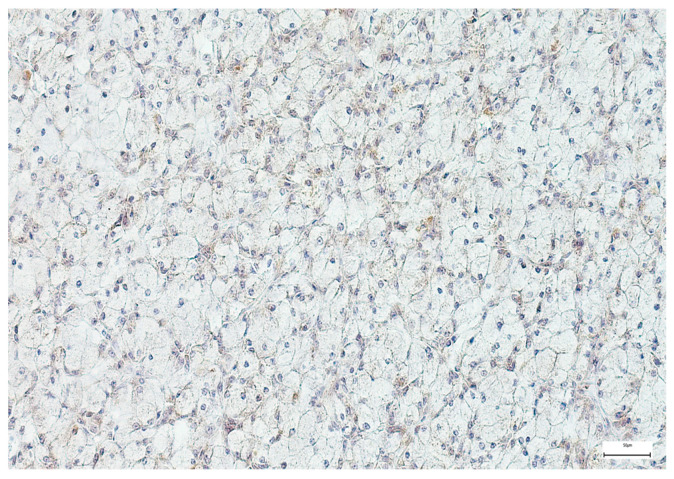
Weak SOX-2 immunoexpression in a salivary gland acinic cell carcinoma (100×).

**Figure 7:**
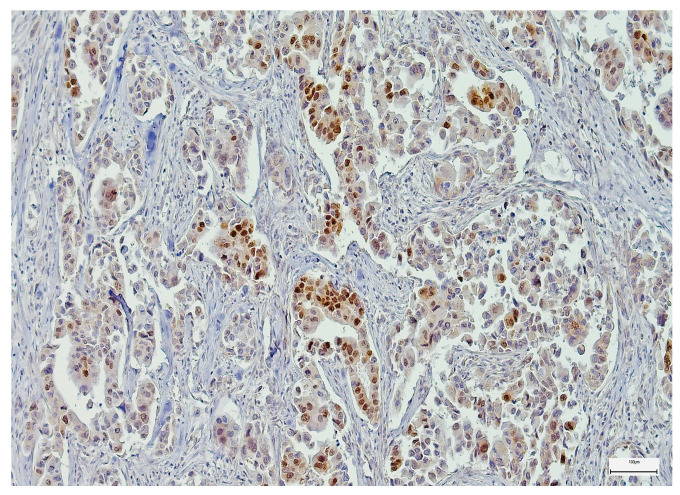
Moderately positive SOX-2 immunoexpression in a salivary duct carcinoma (100×).

**Figure 8:**
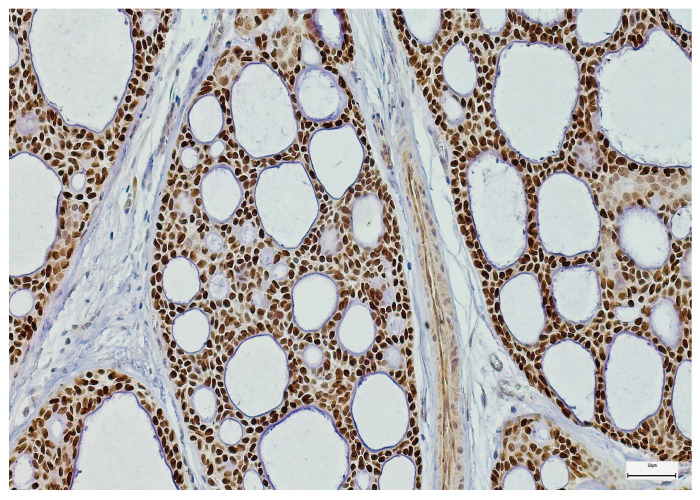
Strong SOX-2 immunoexpression in a salivary gland adenoid cystic carcinoma (100×).

SOX-2 expression was detected mainly in the nucleus of the epithelial MSGT cells in all the examined cases, with variable intensity and distribution in the tumor mass (Figure 6, Figure 7 and Figure 8). In terms of intensity, a weak (1+) SOX-2 staining was detected in 15 cases (14.42%), while moderate (2+) and strong (3+) SOX-2 staining was found in 54 (51.92%) and 35 cases (33.66%), respectively. SOX-2 negative expression was not observed in any tumor. Based on the applied scoring system, low SOX-2 expression was detected in 70 cases (67.30%), while high SOX-2 expression was registered in the other 34 cases (32.70%) of our study group.

**Figure 9:**
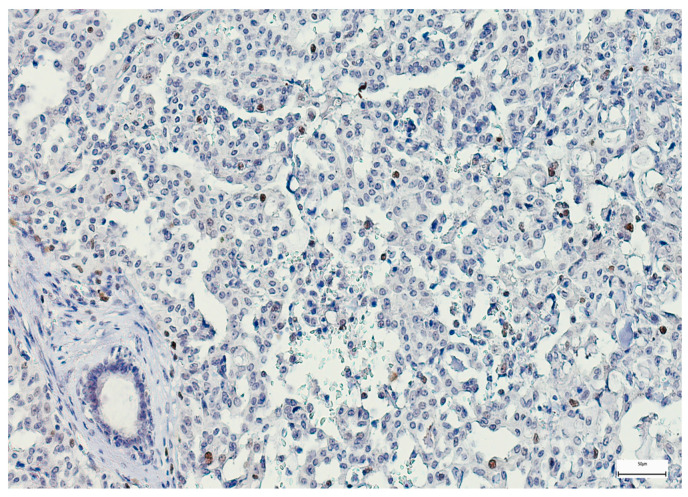
Weak EZH-2 immunoexpression in a salivary gland adenocarcinoma NOS (100×).

**Figure 10:**
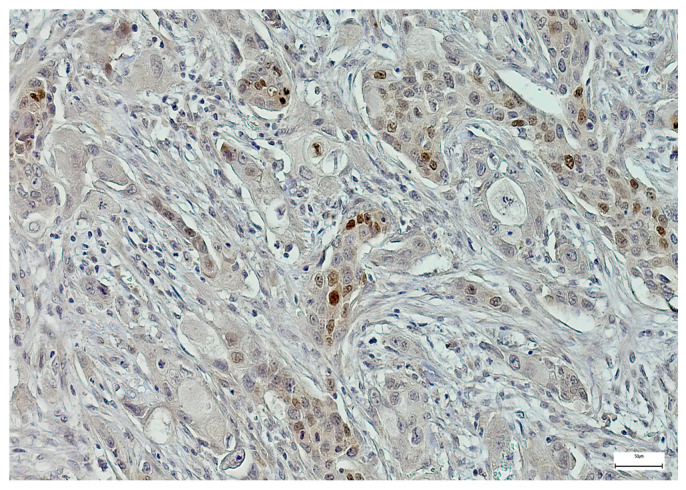
Moderately positive EZH-2 immunoexpression in a mucoepidermoid carcinoma (200×).

**Figure 11:**
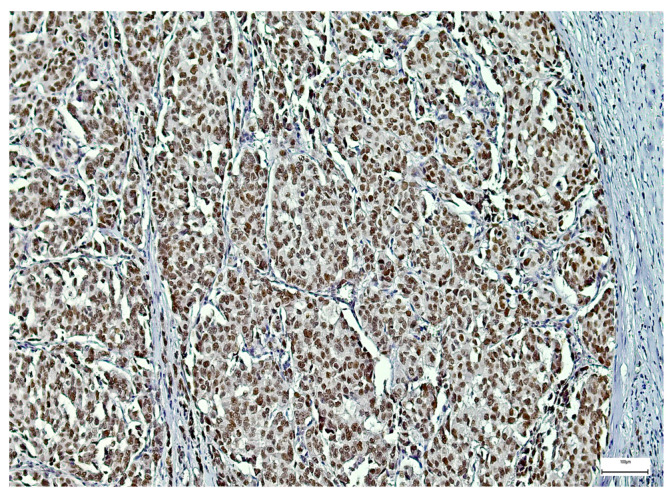
Strong EZH-2 immunoexpression in a salivary duct carcinoma (100×).

EZH-2 expression also exhibited a nuclear pattern in the epithelial tumor cells of the analyzed MSGT cases. Regarding the reaction intensity, most cases exhibited a moderate (2+) EZH-2 expression (*n* = 56 cases; 53.85%), followed by a strong and weak EZH-2 expression (*n* = 42 cases, 40.38%, and *n* = 6 cases, 5.77%, respectively) (Figure 9, Figure 10 and Figure 11). Following the scoring system used in our study, low EZH-2 expression was observed in 45 cases (43.27%), while high EZH-2 expression was detected in 59 cases (56.73%).

### 3.3. Correlation Between SOX-2 and EZH-2 Immunoexpression and Pathological Characteristics

According to the scoring system, high SOX-2 expression was significantly associated with LY, pT stage, histological tumor type, and tumor grading (*p* = 0.003, *p* = 0.010, *p* = 0.003, and *p* = 0.037, respectively). The strength of the association between SOX-2 expression and the pathological characteristics was moderate for pT stage and histological tumor type (V = 0.329) and weak for tumor grading and LY (V = 0.252 and V = 0.289, respectively) (Table 4).

The statistical analysis of the association between EZH-2 expression and the pathological characteristics showed that high EZH-2 expression was significantly associated with PnI, VI, LY, tumor grading, and pENE (*p* < 0.001, *p* = 0.038, *p* = 0.001, *p* = 0.002, and *p* = 0.018, respectively). The strength of the association corresponded to a moderate effect size in PnI, LY, and tumor grading (V = 0.410, V = 0.339, and V = 0.347), and a weak effect size in VI and pENE (V = 0.203 and V = 0.232, respectively). The associations between the pathological characteristics and SOX-2 and EZH-2 expressions are summarized in Table 4.

### 3.4. Correlation Between SOX-2 and EZH-2 Expression and Survival

**Figure 12:**
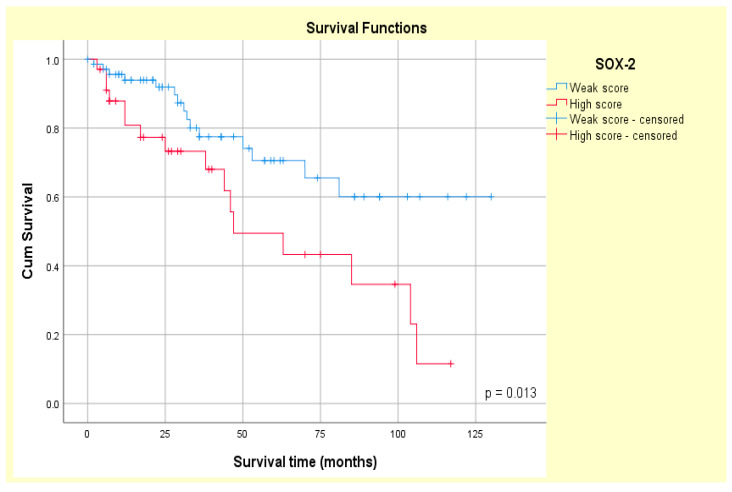
Kaplan–Meier survival analysis according to SOX-2 expression in primary epithelial MSGTs.

**Figure 13:**
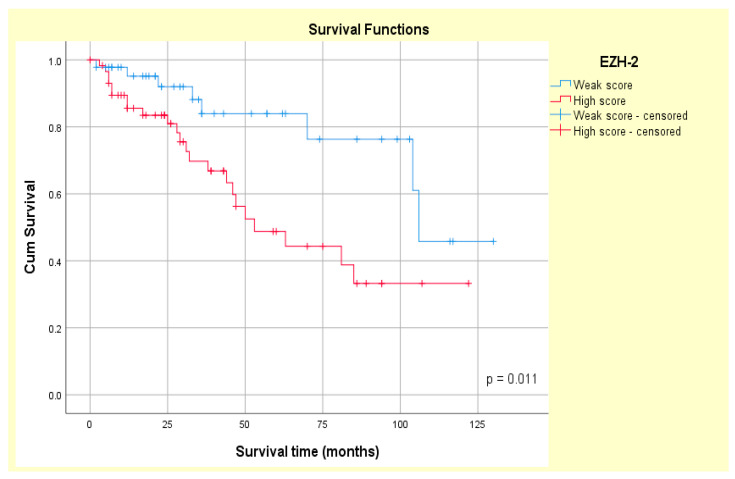
Kaplan–Meier survival analysis according to EZH-2 expression in primary epithelial MSGTs.

Patients exhibiting high SOX-2 tumor expression experienced a shorter median OS (62.58 ± 8.48 months) than those with low SOX-2 tumor expression (95.004 ± 7.46 months). Median OS was 67.76 ± 7.42 months in patients with high EZH-2 expression and 100.74 ± 8.25 months in patients with low EZH-2 expression. The Kaplan–Meier curves revealed that both SOX-2 and EZH-2 tumor overexpressions were associated with poor patient survival in our study group (log-rank Mantel–Cox, *p* = 0.013 and *p* = 0.011, respectively) (Figure 12 and Figure 13).

### 3.5. Prognostic Significance of SOX-2 and EZH-2 and Other Pathological Features

The univariate Cox regression provided convincing evidence for the prognostic value of both SOX-2 and EZH-2 markers (HR = 2.373, *p* = 0.016 and HR = 2.746, *p* = 0.015, respectively) in our study group. Supplementally, EZH-2 tumor overexpression was associated with a higher hazard of death (HR = 2.731, *p* = 0.017) compared with high SOX-2 tumor expression (HR = 2.321, *p* = 0.020), based on multivariate Cox analysis (Table 5).

Regarding the assessment of the pathological features related to primary epithelial MGSTs recommended by the WHO Classification for salivary gland malignancies and AJCC9, the univariate Cox regression analysis showed that pT, LY, PnI, residual tumor, grade, and histological type were significantly associated with OS. However, in multivariate analysis, only pT (HR = 1.826, *p* = 0.019), LY (HR = 0.318, *p* = 0.007), and tumor grade (HR = 0.505; *p* = 0.021) were identified as independent predictors of poor survival (Table 6).

## 4. Discussions

MGSTs are rare tumors with variable geographical distribution in the general population, registering a mortality rate of 0.29 per 100,000 in 2020 [29]. Although comprehensive studies have been conducted regarding etiopathogenesis and MGST prevention approaches, their global incidence and mortality are expected to increase by 50% and 60%, respectively, by 2040 [29]. Moreover, the lowest 5-year relative survival, of 52% (95% CI, 50–54%), is registered in Eastern Europe, compared to a better survival rate, of 74% (95% CI, 71–78%), in the Nordic European countries [2].

These conditions predominantly affect men and are associated with a higher overall incidence in patients over 70 years of age [29]. These data are consistent with our results, showing a slight male predominance, with a higher incidence of cases in the 7th decade (*n* = 30.77%), followed by the 8th and 6th decades of life. However, a multicenter study conducted on a large cohort yielded contrasting results, showing a slight female predominance (53%) among MGST patients [30]. Moreover, women had a lower mean age at diagnosis compared to men (54.4 vs. 57.4 years) [30], in contrast to our findings. These inconsistent findings may be attributed to the influence of hormonal and environmental factors, as well as to the specific genetic characteristics of the population in our geographical area. Furthermore, these contradictory findings are also reflected in the reported location of these lesions, with variations between studies depending on the analyzed geographical area and the size of the study cohort.

Tobacco smoking, radiation, alcohol abuse, and variable viral infections, such as cytomegalovirus (CMV), Epstein–Barr virus (EBV), and human papillomavirus (HPV) infections, are listed as the most significant environmental risk factors in MGST carcinogenesis [23,31,32]. Due to their potential to induce structural genomic changes, smoking and alcohol abuse are associated with a 2-fold and 2.5-fold increased risk of salivary gland malignancies, respectively [23,33]. This finding is also supported by our results, showing a large proportion of MSGT patients as chronic alcohol consumers (68.27%) and current or former smokers (31.73% and 30.77%, respectively), which is in agreement with the reports of previous studies [23,31].

Additionally, obesity has been consistently associated with the development of various types of cancer [34,35]. However, the results regarding the association between salivary gland malignancies and obesity are controversial. Thus, no association between BMI and MSGT occurrence has yet been reported [36,37], and the data are consistent with our observations, with obesity registered in only 18.27% of cases. However, an association between obesity and adenoid cystic carcinoma has been recorded in another recent report [38]. Consequently, further extensive research is needed to characterize the molecular mechanisms of salivary gland carcinogenesis and the influence of environmental factors, particularly obesity, on this process.

Although a substantial proportion of tumors arising in the parotid gland are benign [39], most tumors involving this salivary gland are malignant [40,41,42,43,44]. Moreover, a very recent study based on the data extracted from the German Centre for Cancer Registry Data (ZfKD) confirms the anatomical distribution of these tumors, with the most frequent primary major salivary gland carcinomas being registered in the parotid glands (77.2%), followed by the submandibular (15.5%) and the sublingual glands [45]. However, a considerable proportion of tumors involving the minor salivary glands are malignant, with reports of 70–90% likelihood of a malignancy being diagnosed in a neoplastic mass arising in these locations [43]. Furthermore, a study conducted on a large cohort of patients revealed that the minor salivary glands are the most frequent location of malignant tumors (47%), followed by parotid (42%), submandibular (10%), and sublingual glands (2%) [30]. Our study also supports the variable distribution of these lesions among the salivary glands. Furthermore, the relatively high rate of primary epithelial malignant tumor masses involving the minor salivary glands (35.5% of cases) highlights the recommendation of a careful clinical evaluation of these anatomical regions, especially considering their possible atypical clinical presentation [46].

Mucoepidermoid and adenoid cystic carcinomas were the most frequent histological types of tumor diagnosed in our study, in agreement with previous reports [30,45,46].

Among the different microscopy findings, high histologic grade, LY, VI, and PnI added to higher pT stage and identification of lymph node metastases have already been correlated with poor prognosis and reduced OS [47,48]. Our overall rate of nodal involvement was 51.92%, which is in agreement with previous studies, ranging between 11.8 and 57.4% rate of lymph node metastases [41,42]. LY, VI, and PnI are also adverse pathological features in MGSTs, being constantly and variably reported in the literature [44,49,50]. For example, PnI occurs in more than 40% of MGSTs [44], a feature comparable to our findings. MSGT patients with PnI exhibited distant metastasis, a higher rate of mortality, and a lower recurrence-free survival (RFS) than those without PnI, according to a recent meta-analysis in MSGT patients [50]. Moreover, MSGTs with PnI are more likely to exhibit lymphovascular invasion, advanced pT and pN, a higher tumor grade, and positive margins [49]. However, in another study, PnI was observed in only 20% of the cases of 72 MSGT patients included in a study group, yet survival parameters were comparable between PnI-positive and PnI-negative cases [51]. The variations among these studies may be related to the differences in sample size, the accuracy of PnI evaluation, and pT-related group structure, mainly considering that these features are more frequently detected in pT3 and pT4 stages. In line with these findings, PnI was not proven as an independent prognostic factor following multivariate analysis in our cohort, in contrast to LY, which retained its independent prognostic significance. This finding may be partially explained by the early tumor stages (pT1–pT2) at diagnosis in a substantial rate of cases (47.12%). Similar to our observations, another study reported that Cox regression analysis demonstrated lymphovascular invasion as the most significant factor correlated with patient survival (*p* = 0.027) [52]. This different behavior further supports the distinct biological roles of LY and PnI in tumor progression, with LY reflecting the early tumor spread and the presence of occult micrometastatic disease, whereas PnI is more closely associated with local tumor cell invasion.

Added to lymph node invasion, pENE is a traditional survival predictor in head and neck cancers [42], given that the 9th AJCC version defines any lymph node metastasis with definitive pENE as pN2, the highest nodal category of MSGTs [25]. However, its impact on patients’ survival is still controversial. Some studies have found that pENE influences only disease-free survival (DFS) and the risk of locoregional recurrence [53,54], while other studies have identified ENE as a significant prognostic factor in MSGT patients [55]. pENE+ has been identified in 22.11% of cases of our study group, registering a lower rate compared to the values reported in other studies, ranging between 27 and 61.5% [42,56]. Additionally, this finding may support the lack of pENE+ significance as an independent prognostic factor in our research. This high variability among studies may also be partly explained by the different sizes of the study cohorts, variable MSGTs’ biological behavior, morphological subtypes of nodal involvement, tumors’ variable propensity to nodal metastasis, and a lack of standardization regarding the minimum lymph node count in microscopy evaluation.

Additionally, the residual margin is another major risk factor of local recurrence, with an incidence between 14.3 and 65.4% and poor OS in MSGTs [57]. These data are in agreement with our observations, with positive margins observed in 54.82% of cases, 13.47% of them being grossly positive margins. However, a recent study on 837 patients with MGSTs reported that residual margins were associated with survival in univariate analysis, but residual margins were not demonstrated as an independent prognostic factor in Cox multivariate analysis, which is analogous to our findings [58]. This observation suggests that the effect of residual margin status may be influenced by other prognostic factors, such as pT and adverse pathological features, including tumor grade. Moreover, the relationship between positive margins and pT advanced stages is generally recognized as an adverse prognostic factor, while adjuvant radiotherapy provides an improved survival in these cases [57]. Although the term “close margin” has not yet been clearly defined, a ‘’watch-and-wait’’ approach appears to be the most commonly recommended strategy in these cases [57].

Tumor grade represents a significant factor associated with poor cancer prognosis, including in MSGTs. In this context, multivariate Cox regression analysis in a recent study including 91 MSGT patients revealed that tumor grade proved to be an independent risk factor for poor survival (HR 2.233; *p* = 0.024), which is consistent with our findings [59]. Higher tumor grade has also been proven to be significant for prognosis, added to pT4 stage, pN2 status, and distant metastasis (M1) [60]. This feature highlights a more aggressive tumor biology and an increased metastatic potential associated with high-grade salivary gland malignancies.

SOX-2 is a transcription factor with a key role in the development of the salivary glands, particularly in the acinar cell lineage [9,10]. Limited and sometimes controversial data from the literature are available regarding SOX-2 expression in MSGTs and its overexpression significance [61]. In this context, an increased SOX-2 expression has been strongly associated with advanced pT stage, distant metastases, higher tumor grade, and poor clinical outcome [6,13,62] or with lesions characterized by the absence of myoepithelial differentiation and aggressive MSGT behavior [63].

However, other studies have reported that SOX-2 overexpression does not show a significant association with tumor location or with the absence of perineural invasion [64,65]. Additionally, SOX-2 expression was higher in MSGT patients with lower pT stage (*p* = 0.067) and pN0 compared to patients with pN+ (*p* = 0.047) in another study [66]. Our observations show a partial agreement with these findings, with an association between SOX-2 overexpression and LY, pT stage, histological tumor type, and grading, potentially expressed by a cancer cell population subgroup that drives tumor growth and invasion.

Contradictory findings are also registered regarding SOX-2 relationship with survival parameters, its overexpression being associated with the lowest OS in patients with extracapsular carcinoma ex pleomorphic adenoma [13] or with 5-year OS and DFS in salivary gland adenoid cystic carcinoma [6]. Additionally, a correlation between an increased SOX-2 expression and a poor local control rate (LCR) (*p* = 0.007) has been observed in another study performed on 31 cases of salivary gland squamous cell carcinoma [67]. In agreement with these reports, a significant association between OS and SOX-2 expression was found in our study group (*p* = 0.013). However, other studies revealed no significant association between SOX-2 high expression and prognostic factors [63,68]. Moreover, no statistically significant association of SOX-2 with OS, RFS, or local control rate was found in a group of patients with carcinoma of the major salivary glands [65].

Taken together, further studies performed on larger groups of patients are required to evaluate SOX-2’s role in MSGTs progression and to corroborate its potential as a diagnostic and prognostic marker in these patients. SOX-2 overexpression also suggests an increased stemness activity and, therefore, it may represent a potential therapeutic target in these oncologic patients.

Currently, data on EZH-2 expression in salivary gland malignancies are very limited, particularly regarding its association with clinicopathological features and survival outcomes. Some studies support that increased EZH-2 expression is associated with higher tumor grade, increased invasive potential, tumor recurrence, and poor survival in salivary gland malignancies [14,20,69]. For example, an increased EZH-2 expression was detected in adenoid cystic carcinomas with a predominantly cribriform growth pattern, local metastases, PnI, and recurrences, compared to pleomorphic adenoma, supporting its potential role in cancer progression [14]. These findings are supported by another report, demonstrating that EZH-2 enhanced expression is predominantly noted in salivary duct carcinoma [21] or in adenoid cystic and mucoepidermoid carcinoma (90% and 80.95%, respectively), compared to pleomorphic adenoma (5.88%) [16]. Furthermore, a significant correlation between advanced N and M stages and increased EZH-2 expression in MSGTs (*p* = 0.005 and *p* < 0.001, respectively) was demonstrated in another study, whereas no significant relationship was found between EZH-2 expression and T stage [21]. Our results comply with these study results regarding the correlation between pathological parameters and EZH-2 expression, considering that a high EZH-2 score is associated with unfavorable pathological parameters, such as PnI, VI, LY, grading, and pENE (*p* < 0.001, *p* = 0.038, *p* = 0.001, *p* = 0.002, and *p* = 0.018, respectively), but is not associated with T stage (*p* = 0.216).

EZH-2 overexpression is also correlated with poor survival (*p* = 0.04) in adenoid cystic carcinoma patients, according to previous reports [20], or with decreased clinical benefit rate (CBR) and objective response rate (ORR) (*p* = 0.007 and *p* = 0.039, respectively) [21]. In this context, our study showed that MGST patients with a tumor with high EZH-2 expression registered a lower OS than patients with a tumor with low EZH-2 expression (*p* = 0.011). EZH-2 and SOX-2 showed prognostic significance (HR = 2.373, *p* = 0.016 and HR = 2.746, *p* = 0.015, respectively) in univariate COX regression analysis in our cohort. Additionally, multivariate Cox analysis showed that EZH-2 tumor overexpression (HR = 2.731, *p* = 0.017, and CI = 95%) was associated with a higher death risk compared with high SOX-2 expression (HR = 2.321, *p* = 0.020, and CI = 95%). Therefore, our results suggest that SOX-2 and EZH-2 play a potential role in unfavorable MSGT prognosis.

Accumulating evidence regarding cancer biology shows that OCT4, NANOG, and SOX-2 enhance stemness features in cancer cells, thereby promoting tumor cell proliferation, metastasis, and therapeutic resistance [17,70]. Additionally, EZH2 promotes tumor growth and progression primarily by a mechanism of tumor suppressor gene epigenetic silencing [17,70]. Moreover, EZH-2 may indirectly regulate this master stemness-associated transcriptional network, including SOX-2 activity modulation [17,70]. EZH-2 also maintains a stem-like phenotype in tumor cells by ALDH1 and CD44 expression modulation, a process that is further regulated by SOX-2 activity [71,72]. Consequently, the EZH-2-SOX-2 axis may provide tumor cells with a growth advantage, an undifferentiated phenotype, and a metastatic potential [73]. As a consequence, developing targeted therapeutic strategies aimed at disrupting the SOX-2-EZH-2 axis may represent an innovative therapeutic strategy in oncologic patients.

Regarding oral malignancies, including salivary gland cancers, no clinical trials exploring the therapeutic potential of SOX-2 inhibition have yet been performed. However, indirect strategies to target SOX-2 in cancer are being explored, such as indirect inhibition by upstream pathways, targeted protein degradation, and epigenetic or translational modulators [74,75,76]. For example, due to its potential role in reducing SOX-2 expression, EGFR tyrosine kinase inhibitors, such as gefitinib, showed an anti-tumor activity in experimental oral cancer models [75]. Moreover, the association between mTORC1/C2 inhibition and class I histone deacetylase may limit the progression and the tumor recurrences of human SCC9, SCC15, SCC25, and CAL27 oral squamous cell carcinoma cell lines in a murine model [74]. The first-in-class SOX-2 degraders, such as SOX-2 bioPROTACs (proteolysis targeting chimeras), also exhibit an anti-tumor role in the A549 xenograft model, opening perspectives of new cancer therapy development, including in MSGTs [76].

Regarding EZH-2-based therapy, GSK343 has been recently evaluated for its anti-tumor activity in the oral squamous cell carcinoma CAL27 cell line, demonstrating that it inhibits tumor cell progression by EZH-/Wnt/β-catenin pathway modulation [22]. Additionally, 3-Deazaneplanocin A (DZNep), another EZH-2 inhibitor, may also inhibit the epithelial–mesenchymal transition and the tumor cell progression by STAT3/VEGFR2 axis regulation in CAL27 cell-derived xenograft head and neck squamous cell carcinoma [77]. The combination of tazemetostat (an EZH2 methyltransferase inhibitor) and pembrolizumab was also evaluated for safety and tolerability in patients diagnosed with metastatic or recurrent squamous cell carcinoma of the head and neck in a phase 1 trial study, aimed to establish the recommended phase 2 doses [78].

Despite advances in deciphering the role of SOX-2 and EZH-2 expression in MSGTs, the intricate interactions between SOX-2 and EZH-2 should be further explored by extensive preclinical and clinical trials, which could lead to the development of targeted, personalized therapies for these oncologic patients.

Notably, SOX-2 and EZH-2 may be considered as potential markers for prognosis in these patients, complementing other prognostic biomarkers that have recently demonstrated clinical relevance in salivary gland malignancies [79,80]. In this context, the neutrophil–lymphocyte ratio (NLR) is a classical biomarker used in MSGT prognostic stratification [79], considering that elevated NLR may help to differentiate between parotid low-grade and high-grade malignant tumors [81], being also associated with advanced tumor stage and poor prognosis [79,80]. Additionally, NLR and the systemic inflammatory response index (SIRI) have been shown to provide accuracy in differentiation of benign vs. malignant lesions [80,82]. Platelet–lymphocyte ratio (PLR) combined with the systemic immune-inflammation index (SII) and SIRI has also been recently evaluated in several studies [79,82]. Accordingly, both SII and SIRI can independently predict OS after MSGT surgery [83], while elevated PLR may also serve as a negative prognostic factor in these patients [79]. Overall, these data highlight the necessity of further studies to validate the prognostic utility of both inflammatory and pathological biomarkers, such as SOX-2 and EZH-2, in MSGT patient management.

Due to its exploratory approach, our study aims to provide an overall picture of SOX-2 and EZH-2 expression in relation to the pathological features and the survival outcomes in the primary epithelial MSGT cohort of patients. Given their low incidence, our results provide a general overview of the tumor localization, the associations with risk factors, and the key pathological MSGT characteristics in the Romanian population. Furthermore, the limited information available, mainly regarding EZH-2 expression in MGSTs, highlights the value of our results. However, there are some limitations to this investigation; first are those related to its exploratory design nature, which requires additional studies on larger cohorts of patients, associated with multiple comparative statistical tests to validate our observations. Moreover, this research was conducted in a single-center cohort, and therefore, further studies on larger study groups, including multicenter cohorts and validation in independent datasets, are needed. Secondly, the non-segregation by morphological subtypes provides only a general overview of SOX-2 and EZH-2 molecular profiles in salivary gland carcinogenesis. Multivariate literature analyses show that MSGT prognosis and outcomes are influenced by a complex interplay between different factors, including pathological characteristics. Accordingly, large, standardized studies on extensive patient cohorts are needed for a better stratification of their impact on survival. Finally, our study results’ partial agreement with other investigations’ findings may be determined by study design and different clinicopathological parameters being taken into consideration.

## 5. Conclusions

The association of SOX-2 and EZH-2 activities may contribute to the acquisition of a cancer-aggressive phenotype, with both molecules being considered as potential novel biomarkers of prognosis and possible therapeutic targets in different types of cancers, including in MSGTs. Their molecular interactions may provide novel insights into the salivary gland carcinogenesis in the near future, although additional studies are necessary to confirm and validate the molecular pathways involved in this process.

Furthermore, EZH-2’s potential complex role as a key regulator of the master stemness-associated transcriptional network may open the perspectives of future development of an innovative therapeutic strategy in MSGT patients. Additional data are required to better characterize the prognostic impact of the key pathological factors, such as LY, residual tumor, and tumor grade in MSGTs, given the current evidence heterogeneity.

## Figures and Tables

**Table 1:** SOX-2 expression scoring system.

SOX-2 Positive SGM Cells Staining Percentage	Score	SOX-2 Positive SGM Cells Staining Intensity	Score	Formula	Category
<5%	0	negative	0	staining intensity score x percentage of positive cells	low expression (0–4)
5–25%	1	weak	1		high expression (6–9)
25–50%	2	moderate	2		
>50%	3	strong	3		

SGM cells—salivary gland malignant cells.

**Table 2:** EZH-2 expression scoring system.

EZH-2 Positive SGM Cells Staining Percentage	Score	EZH-2 Positive SGM Cells Staining Intensity	Score	Formula	Category
<5%	0	negative	0	staining intensity score x percentage of positive cells	low expression (0–4)
5–10%	1	weak	1		high expression (6–9)
11–50%	2	moderate	2		
>50%	3	strong	3		

SGM cells—salivary gland malignant cells.

**Table 3:** Baseline data of patients with primary epithelial MSGTs.

Epidemiological and Pathological Characteristics		*n* (%)
**Gender**	women	50 (48.07%)
	men	54 (51.93%)
**Age (years)**	11–19	3 (2.89%)
	20–29	1 (0.96%)
	30–39	10 (9.61%)
	40–49	12 (11.53%)
	50–59	14 (13.47%)
	60–69	32 (30.77%)
	70–79	27 (25.97%)
	80–89	5 (4.80%)
**Smoker status**	non-smoker	39 (37.50%)
	former smoker	32 (30.77%)
	current smoker	33 (31.73%)
**Alcohol consumption**	non-drinker	33 (31.73%)
	drinker	71 (68.27%)
**BMI**	normal weight	52 (50%)
	overweight	33 (31.73%)
	obesity	19 (18.27%)
**Tumor location**	right parotid gland	32 (30.76%)
	left parotid gland	17 (16.35%)
	submandibular glands	14 (13.47%)
	sublingual glands	4 (3.83%)
	palatine glands	22 (21.15%)
	buccal glands	11 (10.60%)
	lingual gland	2 (1.92%)
	labial glands	2 (1.92%)
**Tumor size (mm)**	mean ± SD	36.53 ± 17.41
	min; max	7; 85
**Histological type**	AdCC	30 (28.84%)
	MEC	30 (28.84%)
	SDC	20 (19.22%)
	AcCC	13 (12.5%)
	AC NOS	11 (10.60%)
**Grading**	low	45 (43.27%)
	intermediate	25 (24.03%)
	high	34 (32.70%)
**pT**	T1	15 (14.42%)
	T2	34 (32.70%)
	T3	39 (37.50%)
	T4	16 (15.38%)
**pN**	pNx	2 (1.92%)
	pN0	48 (46.16%)
	pN1	18 (17.31%)
	pN2	36 (34.61%)
**LY**	negative	61 (58.65%)
	positive	43 (41.35%)
**VI**	negative	67 (64.42%)
	positive	37 (35.58%)
**PnI**	negative	43 (41.35%)
	positive	61 (58.56%)
**pENE**	negative	81 (77.89%)
	positive	23 (22.11%)
**EPE**	negative	64 (61.53%)
	positive	40 (38.47%)
**Residual tumor**	R0	47 (45.18%)
	R1	43 (41.35%)
	R2	14 (13.47%)
**Satus**	survivor	73 (70.20%)
	non-survivor	31 (29.80%)

AdCC—adenoid cystic carcinoma; AcCC—acinic cell carcinoma; LY—lymphatic permeation/invasion; AC NOS—adenocarcinoma not otherwise specified; MEC—mucoepidermoid carcinoma; pENE—pathological extranodal extension; EPE—extraparenchymal extension; max—maximum; min—minimum; pN—pathological regional lymph node metastasis; PnI—perineural invasion; pT—pathological tumor stage; R0—complete resection; R1—microscopically positive margin, and R2—grossly positive margins; SDC—salivary duct carcinoma; VI—vascular invasion.

**Table 4:** Relationships between SOX-2 and EZH-2 scoring and pathological characteristics of epithelial MSGTs.

Pathological Parameter	SOX-2 Expression		*p*	*V*	EZH-2 Expression		*p*	*V*
	Low *n*; (%)	High *n*; (%)			Low *n*; (%)	High *n*; (%)		
**pT**			0.010	0.329			0.216	0.207
T1	6 (5.80%)	9 (8.66%)			8 (7.68%)	7 (6.72%)		
T2	24 (23.08%)	10 (9.61%)			18 (17.31%)	16 (15.38%)		
T3	32 (30.77%)	7 (6.72%)			15 (14.43%)	24 (23.08%)		
T4	8 (7.68%)	8 (7.68%)			4 (3.83%)	12 (11.56%)		
**pN**			0.626	0.130			0.144	0.228
pN0	30 (28.84%)	18 (17.31%)			25 (24.03%)	23 (22.11%)		
pN1	14 (13.47%)	4 (3.83%)			9 (8.66%)	9 (8.66%)		
pN2	25 (24.03%)	11 (10.60%)			10 (9.61%)	26 (25%)		
pNx	1 (0.96%)	1 (0.96%)			1 (0.96%)	1 (0.96%)		
**Histological type**			0.003	0.392			0.085	0.281
AdCC	24 (23.08%)	6 (5.80%)			9 (8.66%)	21 (20.19%)		
MEC	16 (15.38%)	14 (13.47%)			16 (15.38%)	14 (13.47%)		
AcCC	13 (12.50%)	0 (0%)			9 (8.66%)	4 (3.83%)		
SDC	13 (12.50%)	7 (6.72%)			8 (7.68%)	12 (11.56%)		
AC NOS	4 (3.83%)	7 (6.72%)			3 (2.89%)	8 (7.68%)		
**Tumor grade**			0.037	0.252			0.002	0.347
low	36 (34.61%)	9 (8.66%)			28 (26.93%)	17 (16.35%)		
intermediate	16 (15.38%)	9 (8.66%)			9 (8.66%)	16 (15.38%)		
high	18 (17.31%)	16 (15.38%)			8 (7.68%)	26 (25%)		
**LY**			0.003	0.289			0.001	0.339
negative	48 (46.16%)	13 (12.50%)			35 (33.66%)	26 (25%)		
positive	22 (21.15%)	21 (20.19%)			10 (9.61%)	33 (31.73%)		
**VI**			0.088	0.167			0.038	0.203
negative	49 (47.12%)	18 (17.31%)			34 (32.67%)	33 (31.73%)		
positive	21 (20.19%)	16 (15.38%)			11 (10.60%)	26 (25.0%)		
**PnI**			0.085	0.169			<0.001	0.410
negative	33 (31.73%)	10 (9.61%)			29 (27.88%)	14 (13.47%)		
positive	37 (35.58%)	24 (23.08%)			16 (15.38%)	45 (43.27%)		
**pENE**			0.794	0.026			0.018	0.232
negative	54 (51.93%)	27 (25.97%)			40 (38.47%)	41 (39.42%)		
positive	16 (15.38%)	7 (6.72%)			5 (4.80%)	18 (17.31%)		
**EPE**			0.974	0.003			0.080	0.172
negative	43 (41.35%)	21 (20.19%)			32 (30.77%)	32 (30.77%)		
positive	27 (25.97%)	13 (12.50%)			13 (12.50%)	27 (25.96%)		
**Residual tumor**			0.055	0.236			0.533	0.110
R0	32 (30.77%)	15 (14.42%)			23 (22.11%)	24 (23.08%)		
R1	25 (24.03%)	18 (17.31%)			16 (15.38%)	27 (25.96%)		
R2	13 (12.50%)	1 (0.96%)			6 (5.80%)	8 (7.68%)		

AdCC—adenoid cystic carcinoma; AcCC—acinic cell carcinoma; LY—lymphatic permeation/invasion; AC NOS—adenocarcinoma not otherwise specified; MEC—mucoepidermoid carcinoma; pENE—pathological extranodal extension; EPE—extraparenchymal extension; pN—pathological regional lymph node metastasis; PnI—perineural invasion; pT—pathological tumor stage; R0—complete resection; R1—microscopically positive margin, and R2—grossly positive margin; SDC—salivary duct carcinoma; VI—vascular invasion. Chi-square test: *p* < 0.05 is significant; V—Cramer’s coefficient.

**Table 5:** Cox regression analysis of SOX-2 and EZH-2 prognostic significance in primary epithelial MSGTs.

	OS			
	Univariate Analysis		Multivariate Analysis	
	HR (95% CI)	*p*-Value	HR (95% CI)	*p*-Value
SOX-2 expression	2.373 (1.172–4.808)	0.016	2.321 (1.144–4.710)	0.020
EZH-2 expression	2.746 (1.220–6.181)	0.015	2.731 (1.197–6.233)	0.017

CI—confidence interval, HR—hazard ratio, OS—overall survival, and significant *p*-value < 0.05.

**Table 6:** Cox regression analysis of main pathological features’ prognostic significance in primary epithelial MSGTs.

	OS			
	Univariate Analysis		Multivariate Analysis	
	HR (95% CI)	*p*-Value	HR (95% CI)	*p*-Value
pT	2.150 (1.397–3.309)	<0.001	1.826 (1.104–3.020)	0.019
pN	1.326 (0.880–1.997)	0.177	-	-
LY	0.273 (0.130–0.575)	0.001	0.318 (0.138–0.732)	0.007
VI	0.730 (0.358–1.489)	0.386	-	-
PnI	2.806 (1.235–6.375)	0.014	1.173 (0.445–3.095)	0.747
pENE	0.701 (0.300–1.641)	0.413	-	-
Residual tumor	1.912 (1.164–3.142)	0.010	1.004 (0.565–1.784)	0.899
Tumor grade	0.477 (0.294–0.774)	0.003	0.505 (0.283–0.901)	0.021
Histological type	1.370 (1.043–1.799)	0.024	1.305 (0.987–1.727)	0.062
EPE	0.770 (0.379–1.565)	0.470	-	-

EPE—extraparenchymal extension; LY—lymphatic permeation/invasion; pENE—pathological extranodal extension; pN—pathological regional lymph node metastasis; PnI—perineural invasion; pT—pathological tumor stage; VI—vascular invasion.

## Data Availability

The original contributions presented in this study are included in the article. Further inquiries can be directed to the corresponding author.
